# Do domestic budgerigars perceive predation risk?

**DOI:** 10.1007/s10071-024-01847-9

**Published:** 2024-03-02

**Authors:** Chang Wang, Xueqi Zhao, Baodan Tao, Jiaqi Peng, Haitao Wang, Jiangping Yu, Longru Jin

**Affiliations:** 1 Jilin Engineering Laboratory for Avian Ecology and Conservation Genetics, School of Life Sciences, Northeast Normal University, Changchun, 130024 China; 2 Jilin Provincial Key Laboratory of Animal Resource Conservation and Utilization, School of Life Sciences, Northeast Normal University, Changchun, 130024 China

**Keywords:** Budgerigar, Predation risk, Feeding behavior, Visual stimuli, Acoustic stimuli

## Abstract

Predation risk may affect the foraging behavior of birds. However, there has been little research on the ability of domestic birds to perceive predation risk and thus adjust their feeding behavior. In this study, we tested whether domestic budgerigars (*Melopsittacus undulatus*) perceived predation risk after the presentation of specimens and sounds of sparrowhawks (*Accipiter nisus*), domestic cats (*Felis catus*), and humans, and whether this in turn influenced their feeding behavior. When exposed to visual or acoustic stimuli, budgerigars showed significantly longer latency to feed under sparrowhawk, domestic cat, and human treatments than with controls. Budgerigars responded more strongly to acoustic stimuli than visual stimuli, and they showed the longest latency to feed and the least number of feeding times in response to sparrowhawk calls. Moreover, budgerigars showed shorter latency to feed and greater numbers of feeding times in response to human voices than to sparrowhawk or domestic cat calls. Our results suggest that domestic budgerigars may identify predation risk through visual or acoustic signals and adjust their feeding behavior accordingly.

**Supplementary Information:**

The online version contains supplementary material available at 10.1007/s10071-024-01847-9.

## Introduction

In nature, predation is the primary cause of death for most animals (Sinclair et al. 2003). Because predators exert strong selection pressure on their prey (Caro 2005), many species have evolved anti-predator behavior to minimize their risk of predation (Lima 1998). However, anti-predator behavior may incur high costs, including energy expenditure, reduced foraging opportunities, and decreased investment in reproduction (Lima 1998; Lima and Bednekoff 1999; Lima and Dill 1990). Animals are capable of trading off effort between foraging needs and predator avoidance depending on the level of threat, as predicted by the threat-sensitivity hypothesis (Helfman 1989). Avian species are often exposed to a higher risk of predation through an increase in foraging time caused by their higher metabolic demands compared to other animals. Thus, avian species should consider tradeoffs between the benefits of foraging and the risk of predation to maximize their fitness (Lima 1998; Lima and Dill 1990). Before this tradeoff can be implemented, avian species must be able to recognize predatory stimuli and accurately assess the risk of predation in the environment.

The majority of avian species primarily use visual and/or acoustic cues to detect predators (Arteaga-Torres et al. 2020; Bartmess-LeVasseur et al. 2010; Billings et al. 2015; Hettena et al. 2014; Smith and Belk 2001). Numerous studies have shown that many avian species can identify the type of predator through visual cues (Duré Ruiz et al. 2018), assess the level of predation threat (Turney and Godin 2014), and adjust their feeding behavior accordingly (Freeberg et al. 2014, 2016). For example, tufted titmice (*Baeolophus bicolor*) were sensitive to the head and body orientation of domestic cat (*Felis catus*) models. They exhibited greater predator avoidance responses when the cat model faced the food (Book and Freeberg 2015). In addition, acoustic cues are essential for some avian species to identify predators. For example, black-capped chickadees (*Poecile atricapillus*) could perceive the level of predation risk according to the type and intensity of predator calls (Congdon et al. 2021). Song sparrows (*Melospiza melodia*) discriminated the calls of Cooper’s hawk (*Accipiter cooperii*), their primary predator, and increased alarm calling during Cooper’s hawk call playback (Akçay et al. 2016).

The domestication process in animals may reduce their fear of humans, and it may also modify their behavior in response to predators (Agnvall and Jensen 2016; Geffroy et al. 2020). Due to the relaxed predation pressure in human-dominated environments, some domestic animal species allocate more time and energy to other important activities to increase their fitness (Jolly and Phillips 2021). For example, domesticated White Leghorn chickens (a breed selected for egg production) have a reduced fear of humans and aerial predators compared to their ancestors, red junglefowl (*Gallus gallus*) (Campler et al. 2009). Although domesticated birds exhibit anti-predator behaviors that are significantly different from those of undomesticated birds (Agnvall and Jensen 2016; Carrete and Tella 2016), some studies conducted on domesticated birds selected for production have shown that they retain some instincts related to recognizing predators. For example, domestic chickens (*Gallus gallus domesticus*) are able to discriminate between aerial and terrestrial predators and produce different alarm sounds (Evans et al. 1993; Gyger et al. 1987; Palleroni et al. 2005), and Mallard ducklings (*Anas platyrhynchos*) have demonstrated an innate capacity to identify different predator types and adjust their anti-predator behavior accordingly (Dessborn et al. 2012). However, less is known about the innate ability of domestic pet birds to recognize predators and whether they have become habituated to humans due to their long-term social interactions with them.

Budgerigars (*Melopsittacus undulatus*) are small-sized scansorial birds native to Australia, and they have a long history of domestication (Polverino et al. 2012). Previous studies have found that budgerigars have acute visual (Chaib et al. 2019) and acoustic perception (Fishbein 2022). For example, a budgerigar was able to spot a conspecific from a distance of 25 m and a bird predator from a distance of 85 m (Chaib et al. 2019), and budgerigars also can extract information from conspecific vocalizations (Fishbein 2022). Therefore, the budgerigar is an ideal species for testing the ability of domestic birds to perceive and recognize predation risk. However, to our knowledge, there are few studies on whether budgerigars can perceive predation risk and make appropriate behavioral decisions.

The main aim of this study was to test whether domestic budgerigars could perceive predation risk and adjust their feeding behavior accordingly. Therefore, we examined the feeding behavior of budgerigars in response to an aerial predator (the sparrowhawk, *Accipiter nisus*), a terrestrial predator (the domestic cat), a potential predator (humans), and controls by conducting dummy and sound playback experiments. We hypothesized that domestic budgerigars would be able to recognize predation risk during feeding. Given that some domestic birds still exhibit the innate ability to recognize predators and that budgerigars have acute visual and acoustic perception as mentioned previously, we predicted that (1) budgerigars would be capable of perceiving predation risk through visual or acoustic cues and thus adjusting their feeding behavior. Because studies have shown that visual cues convey higher certainty of information on predation risk than acoustic cues (Akçay et al. 2016), we predicted that (2) budgerigars would exhibit a greater response to the appearance than to the sounds of predators. Finally, based on humans as the main suppliers of food resources to budgerigars during long-term domestication, we predicted that (3) budgerigars may have become habituated to humans and would show relatively more feeding behavior when facing humans.

## Materials and methods

### Subjects

Adult male budgerigars (*N* = 33) were used as subjects in this study. The birds were purchased from a local pet market, had the same rearing history, and none of them had been previously involved in any experiments. Each bird was marked randomly with a unique numbered metallic leg band. All budgerigars were housed in an indoor feeding chamber, where every individual was housed alone in a cage (90 cm × 40 cm × 50 cm) containing a food box, a water box, and a standing pole. We provided daylight lighting from 7:00 AM to 7:00 PM (12 h:12 h diurnal cycle), a temperature of 20–23 °C, and a relative humidity of 50–60%. The budgerigars were given grain seeds and drinking water at 12:00 AM daily and were regularly supplemented with fresh vegetables, fruits, and parrot nourishment pills to maintain balanced nutrition. All the birds were raised by the same experimenter who always wore the same type of clothes when interacting with them.

### Experimental design

This study was conducted from August to December 2022. All experiments were conducted in a behavior observation chamber (4.0 m × 2.4 m × 2.3 m) containing a birdcage (90 cm × 40 cm × 50 cm) as well as a removable camera (HDR-PJ675; Sony, Shanghai, China) that used to record the behavior of the subjects. A food box and a standing pole were placed inside the birdcage. During the experiments, we could observe the progress in real time through an external monitor placed outside the chamber. The experiments were conducted between 9:00 AM and 12:00 AM. The subject was randomly chosen before the experiment and the food box in its feeding cage was removed approximately 2 h before the beginning of the experiment, with an ad libitum water supply being maintained, so that the bird was slightly starved during the experiment. We used a small birdcage (28 cm × 18 cm × 14 cm) to transfer the subject from the feeding chamber to the birdcage in the behavior observation lab and then left the lab. Each experiment consisted of three periods: a 2-min pre-trial adaptation period, a 3-min predator visual or acoustic stimulation period, and a 2-min observation period after the stimulation. After the experiment, the subject was put back into the birdcage in the feeding chamber and fed at a random time during a 0–2 h period to avoid the subject recognizing the rule of feeding and thus affecting the experimental results.

In this study, an aerial predator (sparrowhawk), a terrestrial predator (domestic cat), and a potential predator (human) were chosen to represent different forms of predation risk. Sparrowhawks are raptors that pose a high level of threat to budgerigars (Carlson et al. 2017). Domestic cats, one of the most popular pets for humans (Courchamp et al. 2003; Crowley et al. 2020; Hunter 2015), not only prey directly upon birds (Murphy et al. 2019) but also increase birds’ fear and stress (Beckerman et al. 2007), indirectly affecting their survival (Fardell et al. 2023; Tryjanowski et al. 2015). In this experiment, we used a sparrowhawk specimen, a domestic cat model made of plush fabric (see the Supplementary Materials), and the sounds of both to test the budgerigars’ responses to natural predators. In addition, birds may associate humans’ presence with the food resources received in a captive environment, thereby reducing responsiveness to humans (Franzone et al. 2022; Ramos et al. 2021). Therefore, we tested the budgerigars’ responses to humans using a human dummy model made of glass-fiber-reinforced plastic wearing cotton clothes (see the Supplementary Materials) and a human voice recording. We used a non-threatening specimen holder (15 cm × 15 cm × 0.5 cm) and background noise as control stimuli. The background noise was recorded in the field and did not involve predator sounds or human voices. Each experimental individual bird was randomly exposed to one kind of visual or acoustic stimulus on each experimental day. The order of stimulus presentation was randomly determined. To ensure the effectiveness of the experiments, an interval of at least 1 day was allowed between two experiments conducted on each individual. The visual dummy experiments and the acoustic playback experiments are described in detail in the following sections.

### Dummy experiments

Stuffed predator specimens and models have been shown to cause birds to respond in a manner similar to how they respond to real predators (Book and Freeberg 2015; Duré Ruiz et al. 2018). We placed the predator specimen or model mentioned above at a distance of 1 m in front of the birdcage. An opaque curtain separated the subject from the predator specimen or model, and we could display or hide the specimen or model by opening or closing the curtain, respectively. During the 2-min adaptation period, the predator specimen or model was hidden behind the curtain, and when the 3-min stimulation period began, the experimenter gently pulled the curtain to expose the subject to the predator specimen or model. At the end of the stimulation period, the curtain was gently closed, and the subject was observed continuously for another 2 min to record the subsequent effects of visual stimulation on feeding behavior. During the experiment, the experimenter was hidden behind the curtain to avoid influencing the subjects.

### Playback experiments

We used Avisoft-SASLab Pro 5.2 (Avisoft Bioacoustics, Berlin, Germany) to choose segments with the lowest levels of background noise based on spectrograms of sparrowhawk calls, domestic cat calls, human voices, and background noise, which we had previously recorded. For each type of sound, we then combined the recordings with natural silence to make a 7-min playback file consisting of a 2-min silent interval (adaptation period) + 3-min of sound (stimulation period) + a 2-min silent interval (follow-up observation period). In the playback experiments, a loudspeaker (M 300; Lang-qin, Shenzhen, China) was placed at a distance of 1 m from the birdcage, and the sound was broadcast at 50 dB SPL at 1 m (Digital Sound-Level Meter 322; Voltcraft, Hirschau, Germany).

### Data collection and analysis

The experimental videos were reviewed to quantify the feeding behavior of each individual from the beginning of the stimulus to the end of the experiment, i.e., the 3-min stimulus period + a 2-min follow-up observation period under different stimulus types. We measured four behavioral variables, i.e., (1) feeding intention: we specified that the subject entering the nearest 1/8 volume area from the food box in the birdcage was considered to have feeding intention; (2) the presence of feeding behavior: the number of individuals exhibiting feeding behavior within specified periods under different stimulus types; (3) latency to feed: the amount of time (s) from the stimulus onset to the presence of the subject’s feeding behavior; and (4) number of feeding times: the number of times each subject took the food.

We performed all statistical analyses in R version 4.2.1 (https://www.r-project.org). We tested the differences in the feeding behavior of budgerigars under different types of predation risks and visual and acoustic stimuli using generalized linear mixed models (GLMMs, glmer in the R package lme4), where the behavioral variables (feeding intention, the presence of feeding behavior, latency to feed, and number of feeding times) were considered dependent variables. The different stimulus types were considered independent variables, and the identification numbers of the budgerigars were considered random variables. To assess the robustness of our results, we calculated both the marginal and conditional *R*^2^ values specific to the models (Nakagawa and Schielzeth 2012). In the case of significant differences in multiple comparisons, we then used the lsmeans function (lsmeans package in R) to make post hoc pairwise comparisons between different experimental groups. Because two-group comparisons after multiple comparisons will increase the probability of type I errors, we used the false discovery rate control in the lsmeans package to adjust* P* values. The significance level was set to 0.05. All data are presented as means ± *SE*.

### Ethical approval

The experimental procedures were approved by the National Animal Research Authority in Northeast Normal University, China (Approval No. NENU-20080416). Behavioral experiments complied with the experimental animal management regulations of the People’s Republic of China (State Scientific and Technological Commission Decree [1988] No. 2) for the ethical treatment of animals and were in line with the ASAB/ABS Guidelines for the Use of Animals in Research. No budgerigars were injured or died during the research.

## Results

### Feeding intention and the presence of feeding behavior

The feeding intention exhibited a binomial distribution, and the frequency of individuals entering the 1/8th volume area closest to the food box was not significantly different between groups during the experiment (GLMMs, *χ*^2^ = 6.14, *df* = 7, *P* = 0.524); in other words, there was no significant difference in the feeding intention of budgerigars among different experimental treatments.

**Fig. 1:**
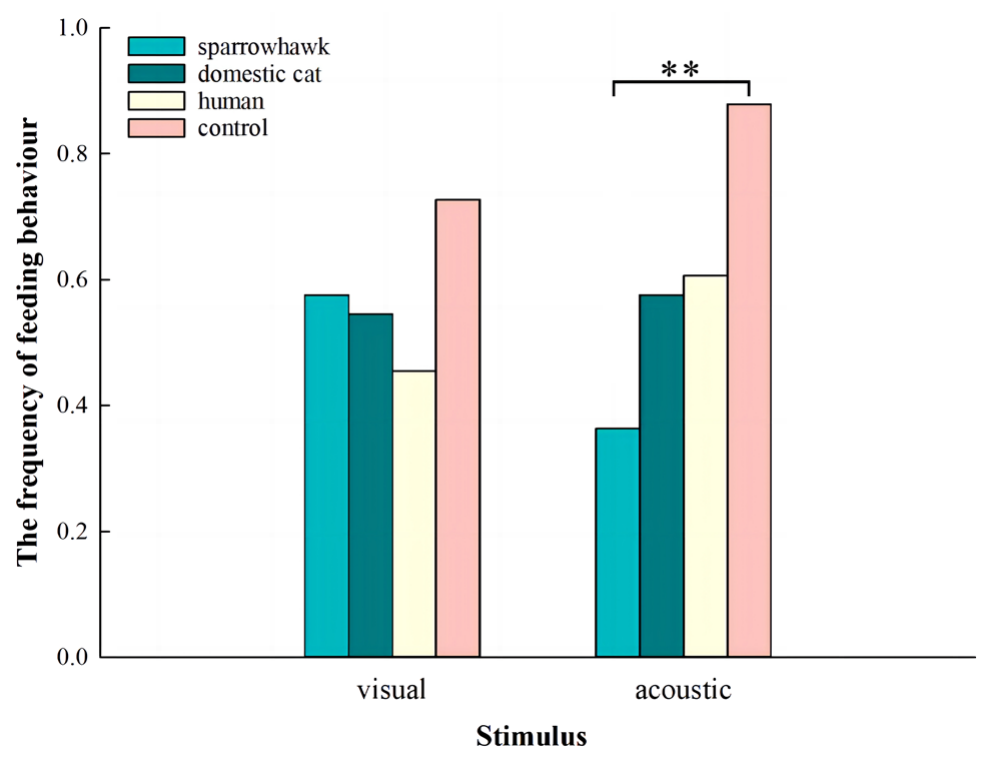
The frequency of feeding behavior in 33 budgerigars in response to different stimulus types. GLMM was used to determine if there was a statistically significant difference between groups under the same stimulus type. **P < 0.01

The frequency of displaying feeding behavior exhibited a binomial distribution, and it under different experimental treatments differed significantly (GLMMs, *χ*^2^ = 22.41, *df* = 7, conditional *R*^2^ = 0.284, marginal *R*^2^ = 0.141, *P* = 0.002; Fig. 1). The frequency of individuals showing feeding behavior was significantly lower in response to sparrowhawk calls than in response to background noise (adjusted *P* = 0.0011, Fig. 1), while there were no significant differences between the other experimental groups (adjusted *P* > 0.05 for all tests).

### Latency to feed

**Fig. 2:**
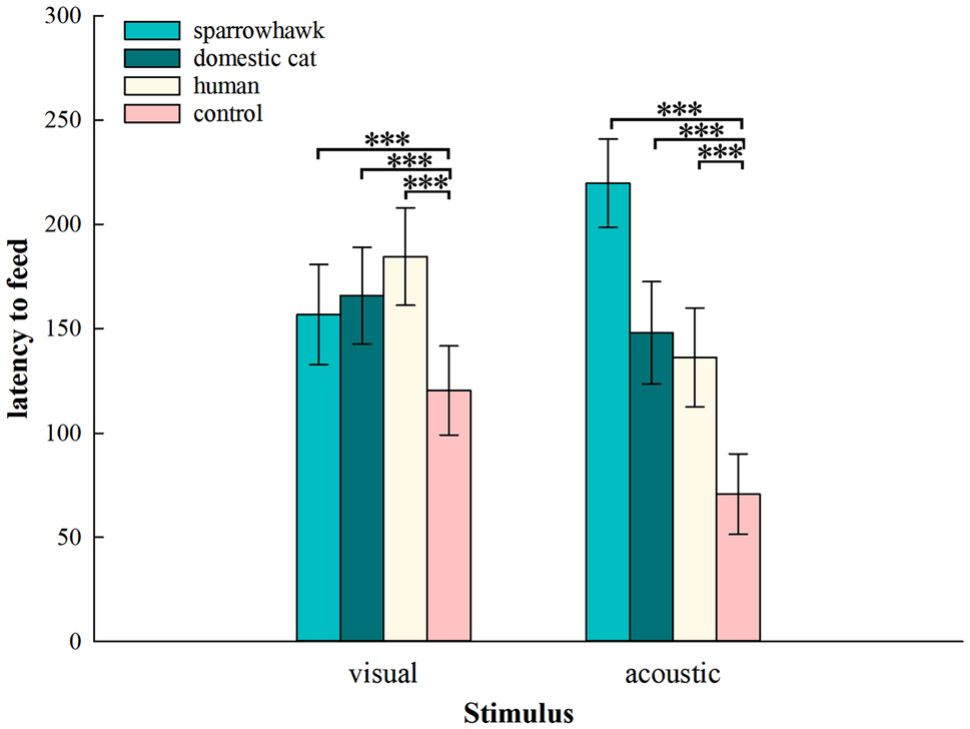
Mean (± *SE*) latency to feed of 33 budgerigars in response to different stimulus types. GLMM was used to determine if there was a statistically significant difference between groups under the same stimulus type. ***P < 0.001

The latency to feed of budgerigars had a Poisson distribution, and it differed significantly among different stimuli (GLMMs, *χ*^2^ = 4492.7, *df* = 7, conditional *R*^2^ = 0.984, marginal *R*^2^ = 0.290, *P* < 0.001; Fig. 2). When budgerigars were exposed to the sparrowhawk, domestic cat, or human in both dummy and playback experiments, latency to feed was significantly longer than with controls (adjusted *P* < 0.001). In dummy experiments, budgerigars responded most strongly to the human dummy model with the longest latency to feed (adjusted *P* < 0.001). In playback experiments, budgerigars exposed to sparrowhawk calls had the longest latency to feed, significantly longer than with domestic cat, human, and control sounds (sparrowhawk calls: 219.67 ± 21.15 s, domestic cat calls: 148.06 ± 24.36 s, human voices: 136.18 ± 23.64 s, and background noise: 70.73 ± 19.33 s, adjusted *P* < 0.001). In addition, the latency to feed of budgerigars exposed to sparrowhawk calls was significantly longer than after exposure to the sparrowhawk specimen (adjusted *P* < 0.001).

### Number of feeding times

**Fig. 3:**
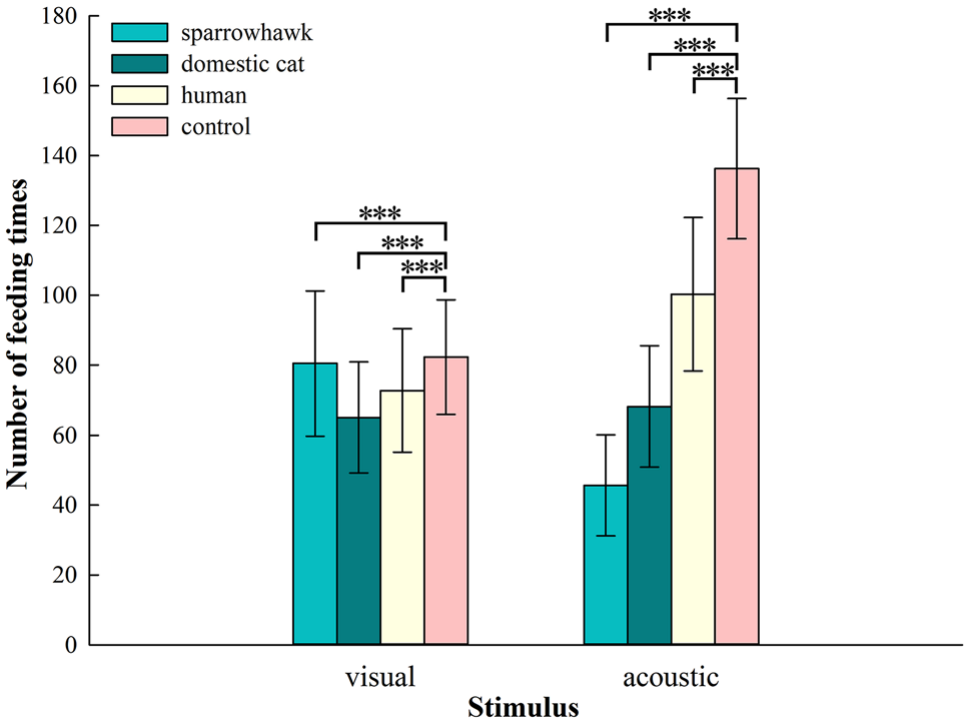
Mean (± *SE*) number of feeding times of 33 budgerigars in response to different stimulus types. GLMM was used to determine if there was a statistically significant difference between group under the same stimulus type. ***P < 0.001

The number of feeding times of budgerigars exhibited a Poisson distribution, and it differed significantly among stimulus types (GLMMs, *χ*^2^ = 2475.5, *df* = 7, conditional *R*^2^ = 0.986, marginal *R*^2^ = 0.141, *P* < 0.001; Fig. 3). In dummy experiments, the number of feeding times was significantly lower when budgerigars were exposed to sparrowhawk, domestic cat, and human models than in the control group (adjusted *P* < 0.001). In playback experiments, the number of feeding times when budgerigars were exposed to sparrowhawk, domestic cat, and human sounds was significantly lower than with control stimuli (adjusted *P* < 0.001). Budgerigars were most sensitive to sparrowhawk calls among all stimulus types, and the number of feeding times when exposed to sparrowhawk calls was significantly lower than that associated with other stimuli (adjusted *P* < 0.001). The number of feeding times when exposed to human voices was significantly higher than with sparrowhawk or domestic cat calls (human voices: 100.27 ± 22.02, sparrowhawk calls: 45.67 ± 14.44, and domestic cat calls: 68.21 ± 17.32, adjusted *P* < 0.001).

## Discussion

Our experiments demonstrated that budgerigars could perceive predation risk through visual or acoustic signals and adjust their feeding behavior according to the level of threat, thus supporting the threat-sensitivity hypothesis (Helfman 1989). This was in line with our first prediction. In this experiment, budgerigars were capable of discriminating the appearance and sound of the aerial predator (sparrowhawk) and the terrestrial predator (domestic cat), responding most strongly to the sparrowhawk calls. In nature, strong predation pressure may drive budgerigars to develop the ability to recognize and respond to predators. As raptors that primarily prey on small birds, sparrowhawks may pose a higher predation threat than domestic cats. Therefore, budgerigars may perceive a higher risk of predation from the sharp and loud calls of sparrowhawks. Previous studies have shown that domestic poultry are able to innately recognize aerial and terrestrial predators (Evans et al. 1993; Gyger et al. 1987; Palleroni et al. 2005). This suggests that although some bird species have undergone a long process of domestication, they likely maintain an innate ability to recognize predators, which may be related to the strong predation selection pressures they faced during their long evolutionary history (Lima and Dill 1990). In contrast, another study showed that the first generation of captive-bred birds rapidly lost their antipredator response and escape abilities compared with wild-caught birds (Carrete and Tella 2016). Therefore, to better understand these inconsistent findings, the ability of more domestic bird species to perceive predation should be explored in the future, which will help us to better understand the selective forces that influence their fitness.

Compared with predator sounds, budgerigars responded more weakly to the motionless predator specimen or model, contrary to our second prediction. The results of a study based on mallard ducklings born in an artificial environment were similar to those of our experiments, i.e., mallard ducklings showed stronger vigilance to the calls of predatory birds, whereas they did not react to still predator specimens (Dessborn et al. 2012). Although birds generally have good eyesight, captive-bred birds without previous experience with predators may not associate still predator specimens with predation risk (Carrete and Tella 2016; Dessborn et al. 2012). In addition, some bird species may have higher detection acuity for moving predators. For example, one study found that blue tits (*Cyanistes caeruleus*) exhibited lower feeding rates in response to moving sparrowhawk specimens than still ones (Carlson et al. 2017). In the future, the response of domesticated birds to moving predators should be further examined.

In line with our third prediction, budgerigars responded less strongly to human voices than to sparrowhawk or domestic cat calls. A possible reason is that budgerigars had lived in a human environment for a long time and might have become habituated to human voices. Thus, they may assess the threat level of humans as being low. A previous study found that scarlet macaws (*Ara macao*) showed lower fear of humans after release and often foraged in close proximity to humans (Brightsmith et al. 2005). In our experiment, the experimenters did not talk to the budgerigars, but before we purchased them at the pet market, they may have been exposed to and become accustomed to human voices, which may have caused their low-intensity response to human voices. However, compared with the sparrowhawk specimen and the domestic cat model, budgerigars showed decreased feeding behavior when confronted with the human dummy model. Although we hypothesized that contact between experimenters and budgerigars during the rearing period could lead to a decrease in their fear of humans, one study showed that visual contact with humans did not affect condition or physiological stress indicators in budgerigars (Price and Lill 2009). In the present experiment, this result was probably due to the unfamiliar human dummy model being chosen instead of the budgerigars’ keeper. Previous studies have shown that red junglefowl chicks that engage in frequent contact (e.g., being hugged and talked to) with an experimenter are more likely to approach the experimenter than a stranger (Rubene and Løvlie 2021). Moreover, predator size affects the intensity of prey anti-predatory behavior, and the prey often must react more quickly to avoid larger predators (Preisser and Orrock 2012; Templeton et al. 2005). Accordingly, budgerigars might have perceived the human dummy model as a novel large predator and showed stronger feeding avoidance responses than toward the smaller sparrowhawk and domestic cat models.

In addition, no significant difference was found in the feeding intention of budgerigars under different forms of predation risk, probably because the budgerigars were in a lightly starved state during the experiment. Most of the subjects approached the food box during the first 2 min of the adaptation period and displayed feeding behavior. When stimulation began, most budgerigars remained in a state of alertness and avoided movements, often staying near the food box, and thus, we judged that they had the intention to take food. Similar results were recorded for other parrot species that also decreased their movements faced with the presentation of a predator-like model (Paulino et al. 2018). At that moment, budgerigars adjusted their feeding behavior according to the level of predation risk. Therefore, the differences in the number of individuals showing feeding behavior, latency to feed, and the number of feeding times under different stimulus types can truly reflect the discrimination of different levels of predation risk by budgerigars.

In this study, there were large differences in feeding behavior among individuals, even under the same stimulus type, a result that may be related to the personality traits of individuals, especially exploration and boldness (Medina-García et al. 2017). Some studies have indicated that personality can influence the exploratory and anti-predator behaviors of birds (Paulino et al. 2018). By increasing exploration, bolder individuals may expose themselves to more risk from predators. In contrast, shy individuals behave more warily in the presence of predators. In addition, antipredator behavior in birds may be sex-linked, with males showing greater vigilance than females in birds with sexual dimorphism (Dávila et al. 2019). Further research is required to test the effects of domestic birds’ personality and sex on their anti-predator behavior.

In summary, we have shown that domestic budgerigars can recognize predation risk through visual or acoustic signals and adjust their feeding behavior accordingly. Budgerigars responded more strongly to predator sounds than appearance, probably because of their weak ability to recognize still predator specimens. In addition, the results of the present study suggest that domestication processes can lead to birds becoming habituated to humans, exhibiting a lower level of alertness to humans. Future studies should determine whether domestication history affects the predator avoidance behavior of birds.

## Supplementary Information

Below is the link to the electronic supplementary material.

Supplementary file1 (XLS 43 KB)

Supplementary file2 (DOC 180 KB)

## Data Availability

The data can be downloaded from the supplementary files and the materials are available from the corresponding authors upon reasonable request.
