# Supplementary material for: Do domestic budgerigars perceive predation risk?
Source: Anim Cogn. 2024 Mar 2;27(1):8. doi: 10.1007/s10071-024-01847-9 (PMC10907484; doi:10.1007/s10071-024-01847-9)
Supplement: Supplementary file 2 — Supplementary file2 (DOC 180 KB) [file 10071_2024_1847_MOESM2_ESM.doc]

**Supplementary materials**


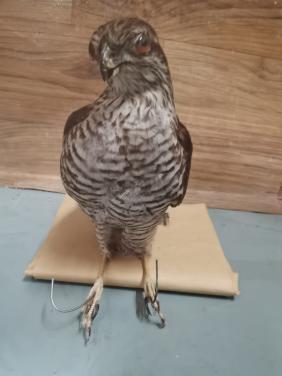

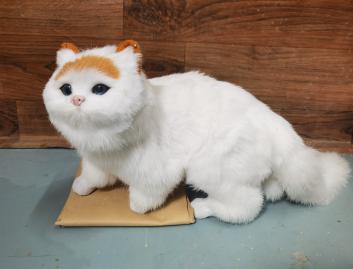

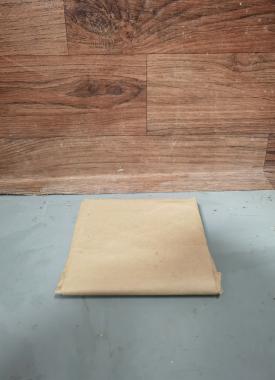

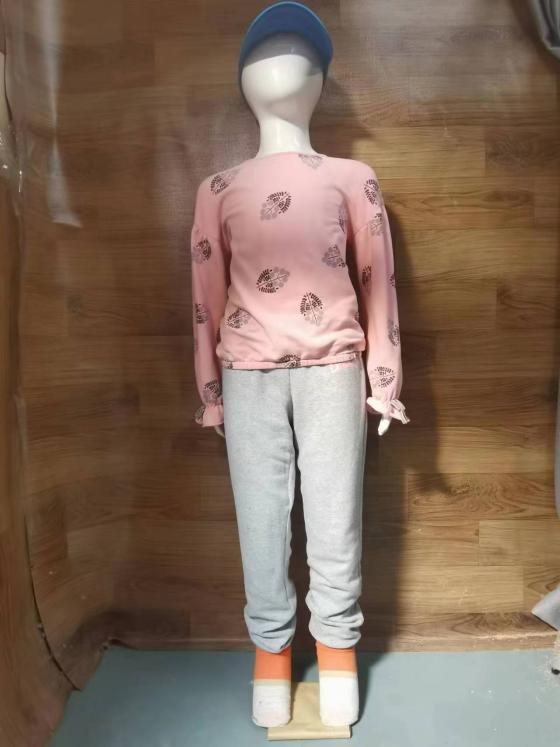


sparrowhawk specimen domestic cat model human dummy model specimen holder
